# Co-Treatment of Caco-2 Cells with Doxorubicin and Gold Nanoparticles Produced from *Cyclopia intermedia* Extracts or Mangiferin Enhances Drug Effects

**DOI:** 10.3390/nano12213918

**Published:** 2022-11-07

**Authors:** Jumoke A. Aboyewa, Nicole R. S. Sibuyi, Mediline Goboza, Lee-Ann Murtz, Oluwafemi O. Oguntibeju, Mervin Meyer

**Affiliations:** 1 Phytomedicine and Phytochemistry Group, Oxidative Stress Research Centre, Department of Biomedical Sciences, Cape Peninsula University of Technology, Bellville 7535, South Africa; 2 DSI/Mintek Nanotechnology Innovation Centre, Biolabels Node, Department of Biotechnology, University of the Western Cape, Bellville 7530, South Africa

**Keywords:** gold nanoparticles, *Cyclopia intermedia*, Mangiferin, nanoparticle uptake, DNA fragmentation, apoptosis, mitochondrial depolarization

## Abstract

Mangiferin (MGF) is a natural and valuable polyphenol found in significant levels in many plant species, including *Cyclopia intermedia* (*C. intermedia*). In a previous study, we synthesized gold nanoparticles (AuNPs) using MGF and a water extract of *C. intermedia* and reported that these AuNPs have very low cytotoxicity toward a human colon cancer (Caco-2) cell line. Although the study also showed that these biogenic AuNPs in combination with doxorubic (DOX) significantly augmented the cytotoxic effects of DOX in Caco-2 cells, the mechanism of the enhanced effect was not fully understood, and it was also not known if other cell lines would be sensitive to this co-treatment. In the present study, we examined the cytotoxicity of the co-treatment in Caski, HeLa, HT-29, KMST-6 and MDA-321 cell lines. Additionally, we investigated the mechanistic effects of this co-treatment in Caco-2 cells using several assays, including the adenosine triphosphate (ATP), the oxidative stress, the mitochondrial depolarization, the colony formation, the APO*Percentage* and the DNA fragmentation assays. We also assessed the intracellular uptake of the biogenic AuNPs. The study showed that the biogenic AuNPs were effectively taken up by the cancer cells, which, in turn, may have enhanced the sensitivity of Caco-2 cells to DOX. Moreover, the combination of the biogenic AuNPs and DOX caused a rapid depletion of ATP levels, increased mitochondrial depolarization, induced apoptosis, reduced the production of reactive oxygen species (ROS) and inhibited the long-term survival of Caco-2 cells. Although the study provided some insight into the mechanism of cytotoxicity induced by the co-treatment, further mechanistic and molecular studies are required to fully elucidate the enhanced anticancer effect of the co-treatment.

## 1. Introduction

The combination of anticancer drugs with natural compounds has emerged as a new and interesting strategy to overcome drug resistance. Several plant extracts and phytochemicals, including polyphenols, alkaloids and carotenoids, are potent chemosensitizers and have the ability to inhibit or even reverse drug resistance [1,2].

Mangiferin (MGF), a natural flavonoid found abundantly in many plant species, has been studied extensively for its therapeutic properties. Several in vitro and in vivo studies have shown evidence for the use of MGF for cancer chemoprevention or in combination with chemotherapeutic drugs as an anticancer treatment [3,4,5]. It is speculated that the anticancer effects of MGF can be attributed to its anti-inflammatory, anti-apoptotic and anti-proliferative properties. Evidently, MGF possesses a unique anticancer property, in that it can reverse drug resistance in doxorubicin (DOX)-resistant cancer cells and is thus recommended as a potential chemosensitizer for DOX therapy. In the work of Louisa et al., MGF was able to sensitize DOX-resistant MCF-7 breast cancer cells to DOX [6]. Takeda et al. also showed that the combination of MGF with either DOX, vincristine or melphalan enhanced the cytotoxic effect of these anticancer drugs in multiple myeloma cells [7]. Considering that MGF has the potential to reverse drug resistance, an opportunity to exploit MGF-rich plants such as *Mangifera indica*, *Salacia chinensis*, *Swertia chirata*, *Hypericum aucheri* and *Cyclopia intermedia* (honeybush, HB) to overcome chemoresistance is currently being explored.

*C. intermedia* is endemic to the fynbos biome of South Africa, and it is widely known for its numerous biological activities, viz., its antioxidant, antimutagenic, antimicrobial and antidiabetic activities [8,9]. Infusions prepared from HB leaves are used for the treatment of infections, coughs, sore throats and colds [8]. Previous studies revealed that HB showed protective effects against skin infection, inflammatory diseases and cancers of the breast, prostate and uterus [8,10]. An evaluation of the bioactive constituents of HB revealed the presence of several phenolic compounds, including MGF [9]. A recent study showed that the cytotoxic effects of DOX were enhanced in Caco-2 cancer cells when used in combination with gold nanoparticles (AuNPs) synthesized using MGF (MGF-AuNPs) [11]. This study also showed that biogenic AuNPs synthesized using water extracts of *C. intermedia* (HB-AuNPs) in combination with DOX have a similar enhanced effect in Caco-2 cells. MGF-AuNPs and HB-AuNPs displayed similar physicochemical characteristics, which strongly suggests that MGF present in HB extracts plays a significant role in the reduction of metal ions to form metallic nanoparticles (MNPs) [11].

The use of plant-mediated biogenic NPs in combination with existing anticancer agents could possibly present an effective treatment regimen in cancer therapy [12]. Mukherjee et al. reported that biogenic AuNPs synthesized using *Peltophorum pterocarpum* and conjugated with DOX (b-AuNPs-PP-Dox) significantly inhibited the proliferation of human lung (A549) and skin melanoma (B16-F10) cancer cells. The study also showed that b-AuNPs-PP-Dox was more effectively taken up and released compared with DOX alone, thus resulting in a significant reduction in tumor growth in C57BL6/J female mice [13].

DOX remains one of the most potent wide-spectrum drugs used in cancer therapy. A study by Wang et al. revealed that DOX induced apoptosis in cardiomyocytes by activating p53 [14]. Additionally, several studies have reported the role of oxidative stress in DOX-induced tumor killing [15,16]. DOX has also been shown to cause damage to mitochondria, which can ultimately also affect respiration. This causes the depletion of adenosine triphosphate (ATP), which subsequently leads to apoptosis [17]. Despite the highly therapeutic effect of DOX, its use is limited because it is associated with cardiotoxicity and nephrotoxicity [18,19]. Several strategies aimed at reducing the toxicity of this drug have been explored, including the development of the nanodrug Doxil. Doxil is the first drug delivery system based on PEGylated (polyethylene glycol coated) liposome technology [20]. Doxil and other liposomal formulations of DOX, including ThermoDox, LipoDox, Myocet and Caelix, were designed to be more tolerable and more effective than free DOX. In cancer patients who have a high risk of cardiac disease, liposomal–DOX formulations offered increased survival compared with free DOX [21].

While the study by Aboyewa et al. demonstrated the potential of MGF-AuNPs and HB-AuNPs to enhance the toxicity of DOX in Caco-2 cells [11], the current study investigated some of the underlying mechanisms of toxicity induced in Caco-2 cells. Additionally, the present study showed that Caski and HT-29 cell lines, but not KMST-6 and MDA-321 cell lines, are similarly sensitive to the effects of the HB-AuNPs and DOX co-treatment. Several bioassays, which include colony formation, ATP, ROS, mitochondrial depolarization and DNA fragmentation assays, were employed to confirm the enhanced effects and mechanisms of the co-treatments on Caco-2 cells. The cellular uptake of MGF-AuNPs and HB-AuNPs in Caco-2 cells was also investigated. The study showed that the biogenic AuNPs were effectively taken up by the cancer cells. Moreover, ATP levels were significantly depleted, while mitochondrial depolarization was increased in Caco-2 cells co-treated with biogenic AuNPs and DOX. In addition, the co-treatment reduced ROS production, inhibited long-term survival and induced cell death through apoptosis in Caco-2 cells. Overall, the current study reported that co-treatment with either MGF-AuNPs and DOX or HB-AuNPs and DOX exhibited similar cytotoxicity in Caco-2 cells. Although the current study provided new insight into understanding the mechanism of the combined effect of biogenic AuNPs and DOX, further mechanistic and molecular studies are recommended to fully understand their synergistic anticancer efficacy.

## 2. Materials and Methods

### 2.1. Synthesis and Characterization of Biogenic MGF-AuNPs and HB-AuNPs

The synthesis and characterization of the biogenic MGF-AuNPs and HB-AuNPs using MGF and water extracts of HB (HBE), respectively, were performed as described previously [11]. Following synthesis, the AuNPs were air-dried and the working concentrations of MGF-AuNPs and HB-AuNPs were prepared by making specified mass-to-volume solutions.

### 2.2. Biogenic AuNPs Cellular Uptake Study

#### Analysis of AuNPs Uptake by Inductively Coupled Plasma—Optical Emission Spectrometry (ICP-OES)

To evaluate the internalization of the biogenic AuNPs by Caco-2 cells, cellular uptake was performed according to published protocols [22] with some modifications. Briefly, cells were cultured in a 12-well plate at a density of 1 × 10^5^ cells/mL and incubated for 24 h at 37 °C in a 5% CO_2_ humidified atmosphere. Afterward, the cells were treated with a single concentration (1000 µg/mL) of either HB-AuNPs or MGF-AuNPs and incubated for 24 h. The cells were harvested via trypsinization and centrifuged at 3000 rpm for 3 min. The resulting cell pellets were washed twice with PBS to remove unbound AuNPs, re-suspended in 2 mL aqua regia (HCl: HNO_3_ in a 3:1 ratio) and digested at 90 °C for 2 h. The digested samples were diluted to 10 mL with 2% HCl (Sigma, St. Louis, MO, USA) and analyzed for gold (AuNP) content using the Varian 710-ES ICP-OES (Santa Clara, CA, USA) at the Department of Chemistry. TraceCERT^®^ (1000 mg/L Au in HCl; Sigma) was used as a standard. The number of AuNPs taken up by cells was calculated based on the initial concentration of AuNPs used for treatment as follows: %AuNP uptake = gold content in test sample/gold content used for treatment * 100% [22]. All treatments were performed in triplicate.

### 2.3. Investigation of the Mechanism of Co-Treatment

#### 2.3.1. Cell Culture and Treatments

The human colon (Caco-2 and HT-29), cervical (Caski and HeLa) and breast (MDA-231) cancer cell lines were purchased from the American Type Culture Collection (Manassas, Virginia, USA), and the non-cancer skin fibroblast (KMST-6) cell line was a generous gift from Prof. Denver Hendricks (Department of Clinical and Laboratory Medicine, University of Cape Town, South Africa). The cells were maintained in Dulbecco’s Modified Eagle Medium (DMEM) (Gibco, Mannheim, Germany) supplemented with 10% FBS (Gibco) and 1% penicillin–streptomycin cocktail (Gibco). Cells were cultured in a humidified 5% CO_2_ incubator at 37 °C. The cells were seeded either in 96-well (1 × 10^5^ cell/mL) or 12-well (2 × 10^5^ cell/mL) microtiter plates and incubated for 24 h at 37 °C in a 5% CO_2_ humidified atmosphere. After 24 h, the medium was replaced with a complete medium containing the respective treatments. The various treatments were MGF-AuNPs-DOX (1000 µg/mL MGF-AuNPs and 1.56 µg/mL DOX), HB-AuNPs-DOX (1000 µg/mL HB-AuNPs and 1.56 µg/mL DOX), HBE (1000 µg/mL), MGF (1000 µg/mL), MGF-AuNPs (1000 µg/mL), HB-AuNPs (1000 µg/mL), Low DOX (1.56 µg/mL) or High DOX (7.8 µg/mL). The negative controls were represented by cells that contained only growth medium without any additional supplements. Cells were treated for 24 h at 37 °C. All experiments were performed in triplicate, repeated thrice.

#### 2.3.2. Cell Viability Assay

The cytotoxic effects of HB-AuNPs-DOX on Caco-2, Caski, HeLa, HT-29, KMST-6 and MDA-231 cells were evaluated using the 3-(4,5-dimethylthiazol-2-yl)-2,5-diphenyltetrazolium bromide (MTT) assay, as described previously [11]. Briefly, cells were individually seeded into sterile 96-well microtiter plates at a density of 1 × 10^5^ cell/mL and incubated at 37 °C in a 5% CO_2_ humidified incubator. Afterward, the growth medium was replaced with 100 µL of HB-AuNPs-DOX prepared in growth medium and further incubated for 24 h. After treatment, 10 µL of MTT reagent (5 mg/mL MTT prepared in medium) was added to each well and further incubated for 3 h. The MTT–medium mixture was replaced with 100 µL of dimethylsulfoxide (Kimix, Cape Town, South Africa), and the absorbance was measured on a PolarStar Omega Plate Reader (BMG Labtech, Ortenberg, Germany) at 570 nm with a reference wavelength of 700 nm. The results were represented as percentage cell viability.

#### 2.3.3. Mitochondrial ToxGlo Assay

The intracellular ATP levels in Caco-2 cells treated for 24 h with MGF-AuNPs-DOX (1000 µg/mL MGF-AuNPs and 1.56 µg/mL DOX), HB-AuNPs-DOX (1000 µg/mL HB-AuNPs and 1.56 µg/mL DOX), HBE (1000 µg/mL), MGF (1000 µg/mL), MGF-AuNPs (1000 µg/mL), HB-AuNPs (1000 µg/mL), and Low DOX (1.56 µg/mL) were assessed using the Mitochondrial ToxGlo assay (Promega, Madison, WI, USA), as described previously [23]. Briefly, cells were seeded in white-walled 96-well microplates and treated for 24 h. Following the treatments, 100 µL of the Mitochondrial ToxGlo reagent (Promega, Madison, WI, USA) was added to each well, and the plate was incubated for 20 min. Thereafter, the ATP levels were quantified by measuring the luminescence on a PolarStar Omega Plate Reader.

#### 2.3.4. Flow Cytometry-Based Bioassays

The mitochondrial depolarization, DNA fragmentation, APO*Percentage* and ROS production assays were used to assess the effects of the treatments on the cells. The assays were performed using flow cytometry as per the kit manufacturer’s guidelines. Caco-2 cells were treated for 24 h; thereafter, both the adherent and detached cells were harvested via gentle trypsinization. All treatments were performed in triplicate. Following the processing of the samples as per the manufacturer’s instructions, the cells were analyzed using a BD Accuri C6 flow cytometer (Erembodegem, Aalst, Belgium). Acquisition was performed in log mode, and approximately 10,000 events per cell sample were acquired. The data were analyzed using Flowjo^TM^ LLC Software v10.6.1, 2019 ( Ashland, OR, USA) and represented as bar graphs.

##### 2.3.4.1. Mitochondrial Depolarization Assay

The effects of the treatments on the mitochondrial membrane potential (ΔΨm) of the cells were investigated using tetramethylrhodamine ethyl ester (TMRE), labeled as described previously [24]. Caco-2 cells were treated as described in Section 2.3.3. Afterward, cells were harvested via gentle trypsinization, washed with PBS and resuspended in media containing 50 nM TMRE (Molecular Probes, Eugene, OR, USA). Following 30 min incubation, the cells were washed, resuspended in PBS and the fluorescence of the cells was assessed using a BD Accuri C6 flow cytometer in the FL-1 channel. Data were acquired as histograms and used to determine the percentage of cells with depolarized mitochondria, which was then presented in bar graphs.

##### 2.3.4.2. DNA Fragmentation Assay

The effects of the treatments on the integrity of the DNA of Caco-2 cells were investigated using the APO-DIRECT^TM^ Kit (BD, Pharmigen, San Diego, CA, USA). The cells were treated as described in Section 2.3.3. The assay was performed according to the manufacturer’s instructions. Following treatment, the cells were stained with FITC-labeled deoxyuridine triphosphates (FITC-dUTP). The cells were analyzed using a BD Accuri C6 flow cytometer. Data were acquired as histograms and used to determine the percentage of cells that were positive for FITC-dUTP, which was then presented in bar graphs.

##### 2.3.4.3. APO*Percentage* Assay

The induction of apoptosis to the Caco-2 cells treated with AuNPs was assessed using the APO*Percentage* assay (Biocolor, County Antrim, UK) and flow cytometry, as described previously [25]. The cells were treated for 24 h with MGF-AuNPs-DOX (1000 µg/mL MGF-AuNPs and 1.56 µg/mL DOX), HB-AuNPs-DOX (1000 µg/mL HB-AuNPs and 1.56 µg/mL DOX), MGF-AuNPs (1000 µg/mL), HB-AuNPs (1000 µg/mL), High DOX (7.8 µg/mL) and Low DOX (1.56 µg/mL). Following treatment, the cells were stained with the APO*Percentage* dye (disodium salt of 3,4,5,6-tetrachloro-2′,4′,5′, 7′-tetraiodofluorescein, TCTF), as previously described. The fluorescence of the cells was analyzed using a BD Accuri C6 flow cytometer in the FL-2 channel [25]. Data were acquired as histograms and used to determine the percentage of cells that were positive for the APO*Percentage* dye, which was then presented in bar graphs.

##### 2.3.4.4. ROS Assay

The effects of the treatments on intracellular ROS generation in Caco-2 cells were evaluated using the 5-(and -6)-chloromethyl-2^1^,7^1^ -dichlorodihydrofluorescein diacetate acetyl ester (CM-H_2_DCFDA) probe (Molecular Probes), following methods previously described [26]. The cells were treated as described in Section 2.3.4.3. After treatment, the cells were gently removed via trypsinization, washed with 1 × PBS, resuspended in 7.5 µM of CM-H_2_DCFDA (prepared in 1 × PBS) and incubated for 30 min at 37 °C, as per the manufacturer’s instructions. Thereafter, the cells were washed with PBS and analyzed using a BD Accuri C6 flow cytometer in the FL-1 channel. Data were acquired as histograms and used to determine the percentage of cells that contained high levels of ROS in comparison with the untreated control. The percentage of cells with high ROS was then presented in bar graphs.

#### 2.3.5. Colony Formation Assay

To investigate whether MGF-AuNPs-DOX or HB-AuNPs-DOX will inhibit the long-term survival of Caco-2 cells, a colony formation assay was performed according to a previously described method [27]. Briefly, Caco-2 cells were seeded in 60 mm Petri dishes at a density of 1 × 10^5^ cell/mL and incubated in a humidified 5% CO_2_ incubator for 24 h. Afterward, the cells were treated for 24 h, as described in Section 2.3.3. Cells were harvested and re-plated in 35 mm dishes at a density of 500 cells/dish and incubated at 37 °C. Their growth was monitored for a period of 14 days, with the growth medium being changed regularly. Cells were fixed and stained with 0.5% crystal violet (Sigma) prepared in 100% methanol (Sigma). A digital camera was used to capture the images of the dishes, and the number of colonies formed was counted using the BioSpectrum Imaging System (UVP, Upland, CA, USA).

### 2.4. Statistical Analysis

Data were expressed as the mean ± standard error of the mean of three experiments performed in triplicate. One-way analysis of variance (ANOVA) was used to determine statistical differences between the groups using the GraphPad^TM^ PRISM version 6 software (San Diego, CA, USA). Samples were considered statistically significant if *p* < 0.05.

## 3. Results and Discussion

### 3.1. Synthesis and Characterization of Biogenic AuNPs

The synthesis and characterization of HB-AuNPs and MGF-AuNPs were documented in our previous study [11]. Overall, the study reported that HB-AuNPs and MGF-AuNPs displayed similar physicochemical properties; a distinct SPR peaks at 540 and 538 nm, hydrodynamic diameters of 66.74 and 65.50 nm, zeta potential values of −23.45 and −27.87 mV and a PDI of 0.571 and 0.432, respectively. HB-AuNPs and MGF-AuNPs were predominantly spherical, with average core sizes of 20 and 26 nm, respectively [11].

### 3.2. Cellular Uptake of Biogenic AuNPs in Caco-2 Cells

**Figure 1:**
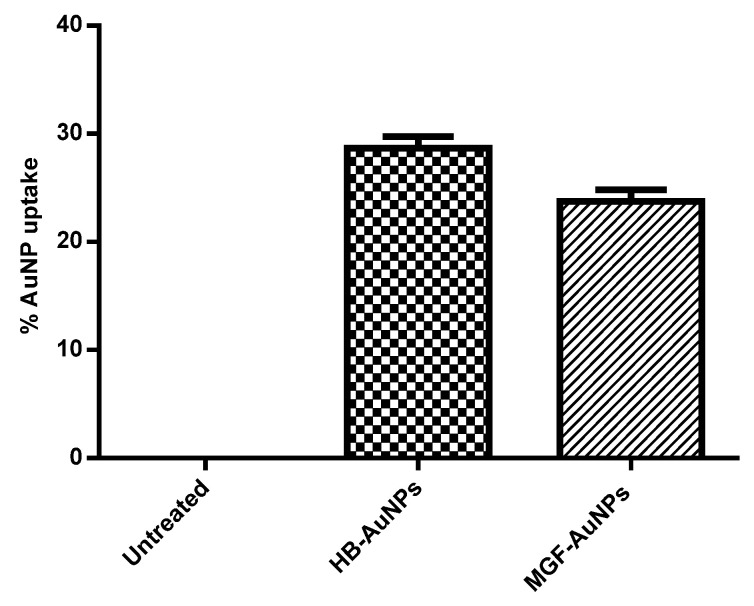
Cellular uptake of AuNPs in Caco-2 cells. Cells were treated for 12 h with HB-AuNPs or MGF-AuNPs, cell lysates were prepared after treatment and Au content within the lysates was determined with ICP-OES. Results are expressed as mean ± standard error of the mean (SEM).

The uptake of MGF-AuNPs and HB-AuNPs in Caco-2 cells was studied with ICP-OES analysis after incubating the cells with AuNPs for 24 h. Figure 1 shows the percentage of gold content recovered from AuNP-treated Caco-2 cells in relation to the total gold content that the cells were exposed to. It was observed that a similar amount of gold was recovered from Caco-2 cells exposed to either MGF-AuNPs or HB-AuNPs. This result indicated that the Caco-2 cells took up approximately 24% of MGF-AuNPs and 29% of HB-AuNPs. Although this study showed that the biogenic AuNPs either bind on the cell surface or are taken up by Caco-2 cells, on their own, they exhibited very low cytotoxicity, as reported previously [11].

### 3.3. Co-Treatment of Cells with HB-AuNPs and DOX

In our previous study, we showed that HB-AuNPs (1000 µg/mL), as well as MGF-AuNPs (1000 µg/mL), exhibited a very low cytotoxic effect in Caco-2 (colon cancer) cells. In addition, we showed that Caco-2 cells were resistant to DOX in low concentrations (1.56–3.125 µg/mL), while higher concentrations (6.25–100 µg/mL) were highly toxic to the cells. Interestingly, when Caco-2 cells were exposed to 1000 µg/mL of either HB-AuNPs or MGF-AuNPs combined with a less-toxic concentration of DOX (1.56 µg/mL), cell viability was significantly reduced to 40% and 70%, respectively [11]. This observation showed that biogenic AuNPs may sensitize Caco-2 cells to DOX and that low concentrations of DOX (which, on its own, is not toxic) become toxic to the cells when used in combination with these biogenic AuNPs.

**Figure 2:**
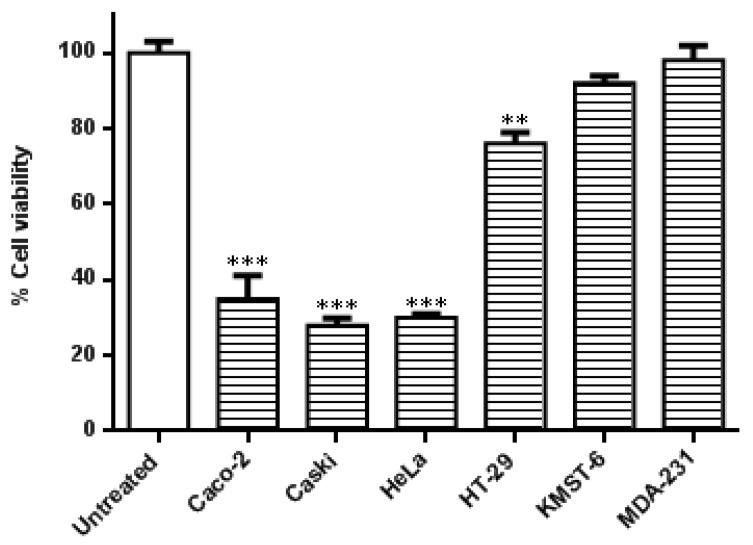
Effects of the co-treatment on the viability of different cell lines. Cells were treated with HB-AuNPs-DOX (1000 µg/mL HB-AuNPs and 1.56 µg/mL DOX) for 24 h, and the viability of the cells was assessed using an MTT assay. Results are expressed as mean ± standard error of the mean (SEM). Data were considered statistically significant if *p* < 0.05, *** *p* < 0.001, ** *p* < 0.01.

In this study, the specificity of the co-treatment with HB-AuNPs and DOX was further evaluated in other cell lines. As shown in Figure 2, the cervical (Caski and HeLa) and the colon (Caco-2 and HT-29) cancer cells were more susceptible to the co-treatment. Caski and HeLa cells, followed by the Caco-2 cells, responded moderately better to the treatment when compared with HT-29 cells. The treatment was not cancer- or cancer-type-specific in the HT-29 cell line which is also a colon cancer cell line showed resistance toward the co-treatment with a viability of 76%. The effects of the HB-AuNPs-DOX co-treatment were negligible in non-cancerous skin fibroblast (KMST-6) and triple-negative breast cancer (MDA-231) cell lines. The difference between the responses of the two colon cancer cell lines (Caco-2 vs. HT-29 cells) suggested that the co-treatment might be targeted at specific molecular pathways, which might not be affected in the less-responsive cell lines, such as HT-29 cells.

### 3.4. The Effects of Co-Treatment with Biogenic AuNPs and DOX

#### 3.4.1. The Effects of Co-Treatment on Cellular ATP Levels

**Figure 3:**
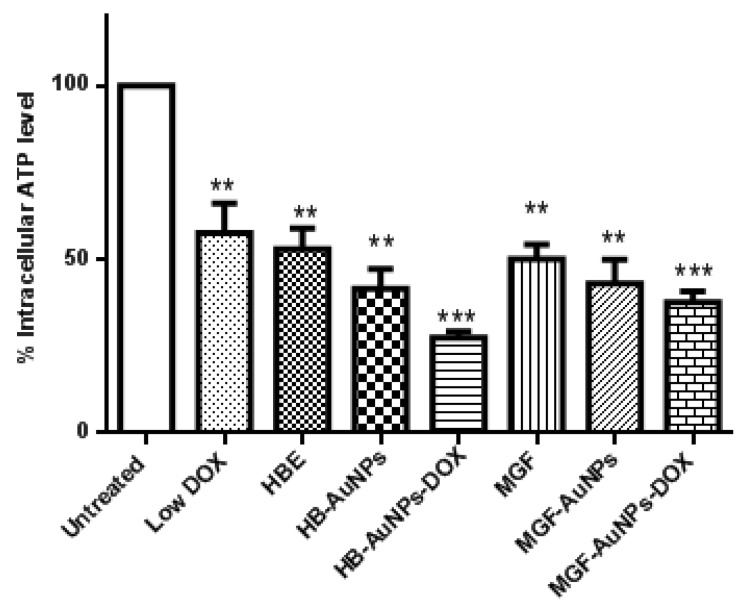
Effect of treatments on intracellular ATP levels in Caco-2 cells. Cells were treated with Low DOX (1.56 µg/mL), MGF (1000 µg/mL), HBE (1000 µg/mL), MGF-AuNPs (1000 µg/mL), HB-AuNPs (1000 µg/mL), MGF-AuNPs-DOX (1000 µg/mL MGF-AuNPs and 1.56 µg/mL DOX) and HB-AuNPs-DOX (1000 µg/mL HB-AuNPs and 1.56 µg/mL DOX). ATP levels were assessed using the Mitochondrial ToxGlo assay. Notes: Results are expressed as mean ± standard error of the mean (SEM). *n* = 3. Data were considered statistically significant if *p* < 0.05, *** *p* < 0.001, ** *p* < 0.01.

In the present study, we investigated intracellular ATP levels in Caco-2 cells that were exposed to HBE, MGF, Low DOX, biogenic AuNPs and a combination of Low DOX and the biogenic AuNPs. Although all treatments resulted in a significant reduction in ATP levels in Caco-2 cells, ATP levels were even more significantly reduced in cells treated with MGF-AuNP-DOX or HB-AuNP-DOX (Figure 3). While ATP levels were 50% and 47% in cells treated with Low DOX and MGF-AuNPs, respectively, ATP levels were further reduced to 35% in cells co-treated with MGF-AuNPs-DOX (MGF-AuNPs and Low DOX). Similarly, treatment with HB-AuNPs-DOX reduced intracellular ATP levels to 28%, compared with 50% for Low DOX alone and 36% for HB-AuNPs alone. It is well known that the main function of the mitochondria is to provide energy in the form of ATP for cell survival, and any pathophysiological alteration in the mitochondrion often leads to impaired mitochondrial functions and, subsequently, results in decreased ATP production and cell death. Therefore, the present study demonstrates that all treatments possibly disrupted mitochondrial function in Caco-2 cells with associated ATP depletion, which subsequently led to cell death. However, the effect was more pronounced in Caco-2 cells treated with either MGF-AuNPs-DOX or HB-AuNPs-DOX. This suggests that the combination of biogenic AuNPs and DOX induced cell death in Caco-2 cells following alteration in mitochondrial function and reduced ATP levels.

#### 3.4.2. The Effect of Co-Treatment on Mitochondrial Function

**Figure 4:**
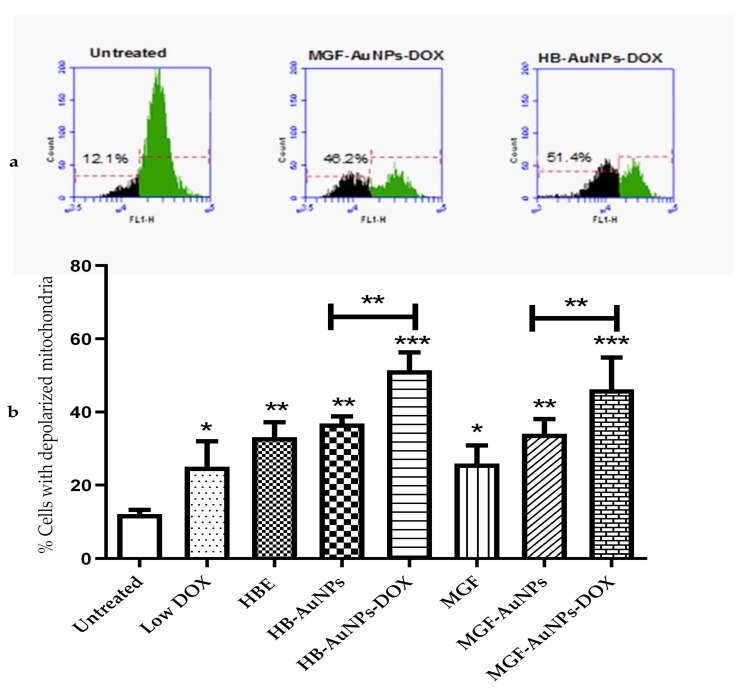
Effect of treatments on mitochondrial function. Caco-2 cells were treated with Low DOX (1.56 µg/mL), MGF (1000 µg/mL), HBE (1000 µg/mL), MGF-AuNPs (1000 µg/mL), HB-AuNPs (1000 µg/mL), MGF-AuNPs-DOX (1000 µg/mL MGF-AuNPs and 1.56 µg/mL DOX) and HB-AuNPs-DOX (1000 µg/mL HB-AuNPs and 1.56 µg/mL DOX). Mitochondrial membrane potential was assessed using a TMRE probe and flow cytometry. (**a**) shows the histograms of the relative fluorescence of cells left untreated, cells treated with MGF-AuNPs-DOX and HB-AuNPs-DOX; green indicates the cell population with normal mitochondrial membrane potential, and black indicates the cell population with reduced mitochondrial membrane potential; indicated on the histograms are the percentage of cells with depolarized mitochondria. (**b**) shows a bar graph, which summarizes the results for all the treatments. Results are expressed as mean ± standard error of the mean (SEM). Data were considered statistically significant if *p* < 0.05, *** *p* < 0.001, ** *p* < 0.01, * *p* < 0.05.

The depolarization of mitochondria has been shown to be associated with impaired mitochondrial functions, including reduced oxidative capacity, enhanced ROS production and reduced ATP production [28], which ultimately leads to cell death. In this study, Caco-2 cells were investigated for changes in mitochondrial membrane potential following a 24 h treatment with HBE, MGF, Low DOX, biogenic AuNPs and a combination of DOX and biogenic AuNPs. The mitochondrial membrane potential in the cells was determined using the TMRE probe. The histograms in Figure 4a show the relative fluorescence of untreated Caco-2 cells compared with cells co-treated with HB-AuNPs-DOX and MGF-AuNPs-DOX. The fluorescence of the cells decreased when the mitochondria become depolarized, as shown for the treated cells (Figure 4a). The percentage of cells with depolarized mitochondria increased from 12.1% for the untreated cells to 48.2% and 51.4% for the MGF-AuNPs-DOX- and HB-AuNPs-DOX-treated cells, respectively. Figure 4b represents a summary of the results and shows that all the treatments resulted in an increase in the percentage of Caco-2 cells with depolarized mitochondria. However, the co-treatments with HB-AuNPs-DOX and MGF-AuNPs-DOX resulted in a higher number of Caco-2 cells with depolarized mitochondria, i.e., 51.4% and 48.2%, respectively. This was a significantly higher percentage of cells with compromised mitochondria compared with the treatment with HB-AuNPs and MGF-AuNPs, which was 37% and 34%, respectively. Treatments with Low DOX and HBE induced mitochondrial depolarization in 25% and 33% of the cells. This shows that the effect of DOX was enhanced almost two-fold when used in combination with biogenic AuNPs compared with DOX alone. These data suggest that biogenic AuNPs enhance the effect of DOX on mitochondrial function. This effect further explains the observed increase in cell death exhibited by Caco-2 cells treated with either MGF-AuNPs-DOX or HB-AuNPs-DOX. Several studies have established that anticancer agents proffer toxicity in cancer cells, which can occur via the induction of ROS and, subsequently, affect mitochondrial function, leading to cell death.

#### 3.4.3. The Apoptotic Effects of Co-Treatment with Biogenic AuNPs and DOX

The cytotoxic assays used in this study indicated that treatment with a combination of biogenic AuNPs and DOX induced cell death in Caco-2 cells. Therefore, some of the hallmarks of apoptosis, i.e., the externalization of the phosphatidyl serine (using the APO*Percentage* assay) and the DNA fragmentation (using the APO-DIRECT^TM^ assay) were also assessed. The APO*Percentage* assay uses a fluorescein dye (TCTF) that becomes trapped within apoptotic cells that are undergoing phosphatidyl serine (PS) externalization and cell membrane blebbing [29] PS is predominantly found in the inner cell membrane but translocates to the outer membrane during apoptosis. PS externalization and cell membrane blebbing are indicators or hallmarks of apoptosis [30]. The APO*Percentage* assay and APO-DIRECT^TM^ assay were performed to evaluate the induction of apoptosis following treatments.

**Figure 5:**
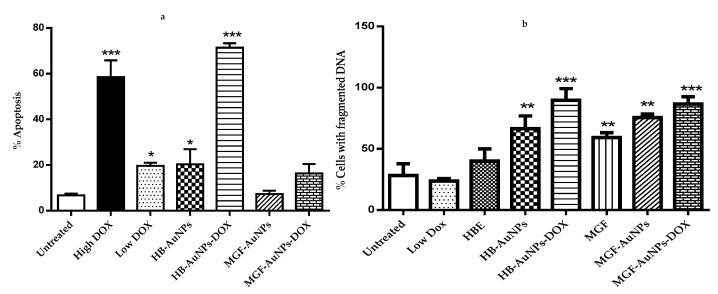
Effect of treatments on apoptosis and DNA fragmentation. Cells were treated with Low DOX (1.56 µg/mL), High DOX (7.8 µg/mL), HBE (1000 µg/mL), MGF (1000 µg/mL), MGF-AuNPs (1000 µg/mL), HB-AuNPs (1000 µg/mL), MGF-AuNPs-DOX (1000 µg/mL MGF-AuNPs and 1.56 µg/mL DOX) and HB-AuNPs-DOX (1000 µg/mL HB-AuNPs and 1.56 µg/mL DOX); (**a**) shows the results of apoptosis assessed using the APOPercentage assay and flow cytometry; (**b**) shows the results of the DNA fragmentation assay assessed using the APO-DIRECT^TM^ assay and flow cytometry. Results are expressed as mean ± standard error of the mean (SEM). Data were considered statistically significant if *p* <0.05, *** *p* < 0.001, ** *p* < 0.01, * *p* < 0.05.

The results in Figure 5a show that only treatments with DOX (1.56 and 7.8 µg/mL), HB-AuNPs and HB-AuNPs-DOX induced significant levels of apoptosis in Caco-2 cells. However, while treatments with HB-AuNPs and 1.56 µg/mL DOX alone induced moderate levels (±20%) of apoptosis, the combined treatment with HB-AuNPs and 1.56 µg/mL DOX (HB-AuNP-DOX) induced very high levels (70%) of apoptosis. This again demonstrated the synergistic effects of the co-treatment and confirmed that the cells die via apoptosis. Surprisingly, the co-treatment with MGF-AuNPs-DOX and 1.56 µg/mL DOX (MGF-AuNP-DOX) did not induce significant levels of apoptosis in Caco-2 cells. However, PS externalization occurred at an early apoptotic event, and it is thus possible that the time it takes for PS externalization is different for these two treatments. That is, PS externalization in cells treated with MGF-AuNP-DOX may occur at an earlier time point. A time-course experiment will resolve this question.

The activation of endonucleases during apoptosis results in the fragmentation of nuclear DNA [31]. In the later steps of cell death, chromosomal DNA is cleaved into smaller fragments through the action of endonucleases. The APO-DIRECT^TM^ assay can be used to label these DNA fragments with FITC-dUTP using the TdT enzyme, which incorporates FITC-dUTP into the 3′-hydroxyl-DNA ends of the DNA fragments found in apoptotic cells [32]. The current study assessed DNA fragmentation in cells treated with HBE, Low DOX (1.56 µg/mL), MGF-AuNPs, HB-AuNPs, MGF-AuNPs-DOX and HB-AuNPs-DOX. Figure 5b shows that all these treatments, except for treatments with HBE and DOX, induced significant levels of apoptosis in Caco-2 cells. However, while treatment with HB-AuNPs alone did induced DNA fragmentation, when used in combination with DOX, the percentage of cells with fragmented DNA was higher. Similarly, while MGF-AuNPs and MGF treatments did induced DNA fragmentation, the percentage of fragmented DNA was higher in cells treated with MGF-AuNPs-DOX. This result also supports the comment made previously that the absence of PS externalization for MGF-AuNP-DOX-treated cells does not mean these cells do not die via apoptosis.

Apoptosis is a natural mechanism adopted by living organisms for the removal of damaged or unwanted cells. It is a highly regulated process that plays a vital role in the pathogenesis of several pathological and physiological processes, including cancer. It has been documented that DOX induces apoptosis by first inhibiting topoisomerase II. This action leads to the generation of free radicals and DNA damage, which subsequently results in cell death [33] An earlier study reported that DOX induced apoptosis in breast (MCF-7) cancer cells following treatment with 4 µM of DOX [34] A study by Wang et al. showed that 0.5 µM of DOX induced apoptosis in human ovarian teratocarcinoma (PA-1) and human breast adenocarcinoma (MCF) cells via caspase-3 activation and DNA fragmentation [14]. However, colorectal cancer cells (CRCs) are inherently resistant to DOX, and higher doses of this drug are, therefore, required to eradicate these cells. On the other hand, these high doses are associated with cardiotoxicity and nephrotoxicity [18,19]. Unfortunately, the mechanism by which these cells develop resistance is not fully known. Xiong and Xiao suggested the activation of the steroid receptor co-activator, while Kubiliūtė et al. reported the involvement of epithelial–mesenchymal transition, cell adhesion and motility [28,35]. Thus, strategies to overcome DOX resistance, as well as restore the sensitivity of cancer cells to DOX, become necessary. A study by Khaleel et al. showed that the combination of resveratrol and didox reversed the DOX resistance of HT-29 cells and decreased the IC_50_ of DOX from 0.88 µM to 0.47 µM [36]. Another study showed that glutathione-stabilized AuNPs were able to sensitize a DOX-resistant OSA D17 (bone cancer) cell line to DOX by overcoming P-glycoprotein-mediated multidrug resistance [37]. In this case, it is believed that AuNPs can surpass the size of the P-gp channel and accumulate, thus preventing drug efflux [37]. The present study strongly suggests that the co-treatment with HB-AuNPs and DOX possibly has multiple targets in the apoptotic pathway, leading to increased cell death in Caco-2 cells.

#### 3.4.4. The Effect of the Biogenic AuNP and DOX Co-Treatment on ROS Levels

Although the elevation of ROS levels appears to be one of the paradigms for NP-mediated toxicity [37], the direct toxicity of NPs without the overproduction of ROS has been reported previously [38] Consequently, high ROS production may not necessarily be required for NP-induced toxicity [39]. On the other hand, DOX induces death in cancer cells by increasing cellular ROS levels. Several studies have shown that DOX-induced cardiotoxicity is mainly caused by ROS-induced cellular damage, which also limits the use of this anticancer drug [40]. Accordingly, the present study also investigated what effect co-treatment with the biogenic AuNPs and DOX had on the ROS levels of Caco-2 cells using the CM-H_2_DCFDA fluorescent probe and flow cytometry.

**Figure 6:**
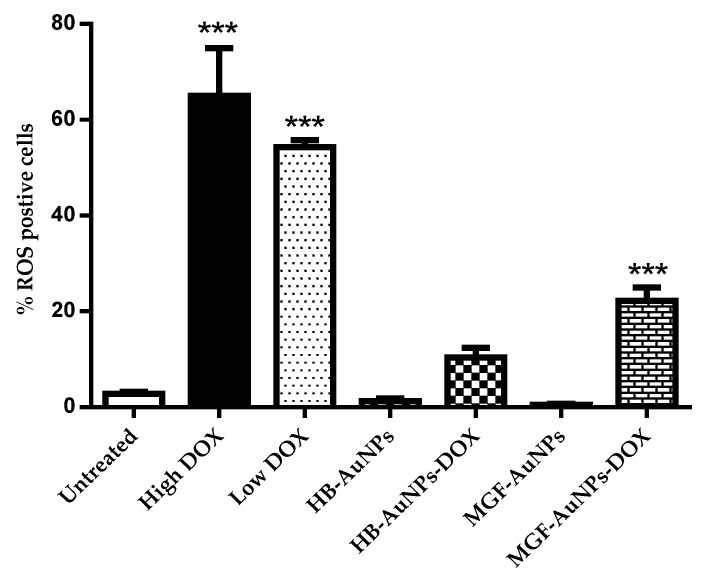
Effect of the treatments on ROS production. Caco-2 cells treated with Low DOX (1.56 µg/mL), High DOX, MGF-AuNPs (1000 µg/mL), HB-AuNPs (1000 µg/mL), MGF-AuNPs-DOX (1000 µg/mL MGF-AuNPs and 1.56 µg/mL DOX) and HB-AuNPs-DOX (1000 µg/mL HB-AuNPs and 1.56 µg/mL DOX) and the level of ROS production was assessed using the CM-H2DCFDA probe and flow cytometry. Results are expressed as mean ± standard error of the mean (SEM). Data were considered statistically significant if *p* < 0.05, *** *p* < 0.001.

Treatments with either 1.56 µg/mL or 7.8 µg/mL DOX induced significantly high levels of ROS production in Caco-2 cells, while treatments with HB-AuNPs and MGF-AuNPs alone did not induce any significant increase in ROS compared with the untreated cells (Figure 6). Interestingly, co-treatments with 1.56 µg/mL of DOX and HB-AuNPs (HB-AuNPs-DOX) or 1.56 µg/mL of DOX and MGF-AuNPs (MGF-AuNPs-DOX) also induced ROS production; the percentage of cells with high ROS levels was lower for the co-treatments when compared with the treatment with Low DOX alone. The increase in ROS production observed in Caco-2 cells treated with DOX was expected and is in agreement with previous studies [41,42]. The results of this study suggest that the biogenic NPs (HB-AuNPs and MGF-AuNPs) counter or reduce ROS production even in the presence of DOX. This further suggests that the toxicity induced by the HB-AuNPs-DOX and MGF-AuNPs-DOX is probably not due to increased ROS production, but rather via some other mechanism. It also suggests that the biogenic NPs protect the cells against ROS-induced cell damage. This is a significant finding, as this may suggest that a co-treatment with biogenic NPs and DOX might be less toxic to cardiomyocytes. However, this will require further investigation.

It is well known that HB contains high concentrations of polyphenols such as hesperidin, MGF, caffeic acid and gallic acid [8]. Polyphenols are powerful antioxidants that scavenge oxygen-derived compounds or prevent the formation of ROS [9]. It is possible that the high nucleophilic properties of the hydroxyl and carbonyl groups in these polyphenols are involved in the capping of metal ions during the formation of biogenic AuNPs and play a role in blocking the formation of oxygen radicals when the AuNPs are taken up by cells.

### 3.5. The Effect of the Biogenic AuNP and DOX Co-Treatment on the Long-Term Survival of Caco-2 Cells

**Figure 7:**
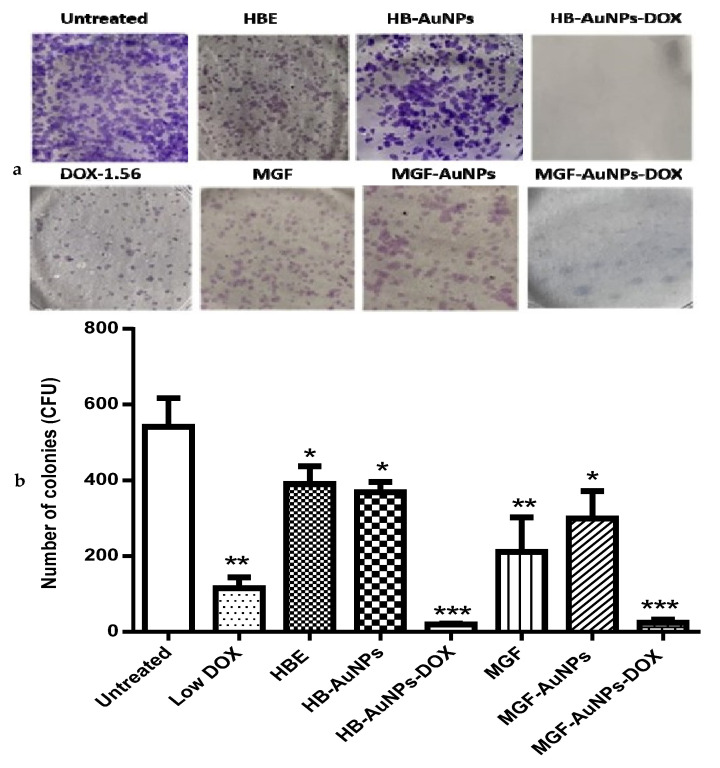
Effects of treatments on the long-term survival of Caco-2 cells. The clonogenic assay was used to assess the effects of Low DOX (1.56 µg/mL), MGF (1000 µg/mL), HBE (1000 µg/mL), MGF-AuNPs (1000 µg/mL), HB-AuNPs (1000 µg/mL), MGF-AuNPs-DOX (1000 µg/mL MGF-AuNPs and 1.56 µg/mL DOX) and HB-AuNPs-DOX (1000 µg/mL HB-AuNPs and 1.56 µg/mL DOX) on the long-term survival of the cells. After a 24 h treatment, cells were cultured in medium without treatments for 14 days. Surviving cells were stained crystal violet; (**a**) shows images of the colonies (in blue) that were present after 14 days; (**b**) shows a summary of the colonies counted. Results are expressed as mean ± standard error of the mean (SEM). Data were considered statistically significant if *p* < 0.05, *** *p* < 0.001, ** *p* < 0.01, * *p* < 0.05.

The clonogenic assay was performed to determine the reproductive viability of Caco-2 cells following treatment with either MGF-AuNPs-DOX or HB-AuNPs-DOX. The assay essentially tests every cell in the population for its ability to undergo unrestricted division [30]. It is also a standard method of assessing the efficacy of radiation or cytotoxic agents in vitro. The assay is based on the ability of a single cell to survive and grow into a colony of at least 50 cells after a short period of exposure to therapeutics [43]. In this study, Caco-2 cells were exposed for 24 h to HBE, MGF, Low DOX, biogenic AuNPs and a combination of DOX and biogenic AuNPs, and then they were allowed to grow for fourteen days, during which time the growth medium was regularly changed. The colonies formed were counted to estimate the differences in cell survival after treatment. As shown in Figure 7, the number of colonies formed following treatment with Low DOX, MGF, MGF-AuNPs, HBE and HB-AuNPs were significantly reduced compared with the untreated control (Figure 7a). The capability of cells to form colonies following treatment with either MGF-AuNPs-DOX or HB-AuNPs-DOX was even more severely affected. The number of colonies formed after treatment with DOX, MGF-AuNPs and HB-AuNPs alone was 115, 299 and 369, respectively; only 24 and 20 colonies were formed after treatment with MGF-AuNPs-DOX and HB-AuNPs-DOX, respectively. Figure 7b shows a picture of the stained colonies. The reduction in colonies formed after co-treating the cells with biogenic AuNPs and DOX is suggestive of the high cytotoxicity and damage caused by the combination therapy. Thus, the co-treatment with either HB-AuNPs and DOX or MGF-AuNPs and DOX has a robust anticancer effect in Caco-2 cells, preventing the recovery of the cells from the treatment. Overall, the combination of biogenic AuNPs and DOX has therapeutic potential for CRC treatment because targeting the clonogenic subset of cancer cells is strongly believed to be essential for successful cancer therapy [30].

## 4. Conclusions

This study demonstrated the potential of using biogenic AuNPs to enhance the efficacy of DOX in vitro. Co-treatment with the biogenic AuNPs and DOX induced selective cytotoxicity in cervical and colon cancer cell lines. In Caco-2 cells, cell death occurred through various mechanisms that suggested that the cells died through programmed cell death (apoptosis), i.e., the externalization of PS, the depletion of ATP levels, mitochondrial membrane depolarization and the induction of DNA fragmentation. Additionally, the co-treatment also inhibited the long-term survival of Caco-2 cells. Together, these findings strongly suggest that these biogenic AuNPs can serve as drug sensitizers when used in combination with chemotherapeutic drugs and enhance their efficacy at a very low dosage. However, cytotoxicity studies in other cancer and non-cancer cells, as well as their exact molecular mechanisms, are recommended to unravel the full potential of the co-treatment therapy.

## Data Availability

The data presented in the study can be requested from the authors.
